# Use of a cholesterol emulsion in Smith-Lemli-Opitz syndrome (SLOS): A single-center observational study, retrospective analysis and structured caregiver interview

**DOI:** 10.1186/s12944-025-02767-4

**Published:** 2025-10-22

**Authors:** Tobias Fischer, Alicia Born, Simone Harmeling, Manfred Fobker, Ulrike Och, Christiane Elpers, Thorsten Marquardt

**Affiliations:** 1 Center for Nutrition and Therapy (NuT), University of Applied Sciences Muenster, Corrensstraße 25, 48149 Muenster, Germany; 2 Department of Pediatrics, University Hospital Muenster, Albert-Schweitzer-Campus 1, 48149 Muenster, Germany; 3 Central Laboratory, University Hospital Muenster, Albert-Schweitzer-Campus 1, 48149 Muenster, Germany; 4 Christian children’s hospital (CKO), Johannisfreiheit 1, 49074 Osnabrück, Germany

**Keywords:** Smith-Lemli-Opitz syndrome, SLO, SLOS, Cholesterol, Cholesterol metabolism deficiency, Cholesterol emulsion, Cholesterol supplementation, Dietetic therapy, Dietary supplement, Metabolic diseases, Congenital metabolic diseases

## Abstract

**Background:**

Smith-Lemli-Opitz syndrome (SLOS) is a rare autosomal recessive disorder of cholesterol biosynthesis. SLOS leads to increased levels of 7-dehydrocholesterol (7-DHC) and decreased levels of total cholesterol (TC). Dietary therapy usually involves supplementation with cholesterol in an oil-based or, less commonly, an aqueous cholesterol suspension. The limited solubility of cholesterol can result in uneven distribution, sedimentation and clumping.

**Methods:**

In seven patients (6 m, 1 f, 1–12 years) the previously administered dose of cholesterol was replaced by a newly developed emulsion and primary parameters TC, 7-DHC, HDL, LDL, vitamin D (25; 1–25), height and weight were determined. In addition, a personal structured interview was conducted with the caregivers of five participants to determine their satisfaction with the product, the care, and the effects on behaviour and health.

**Results:**

One patient was excluded due to non-compliance (*N* = 6). Before the intervention, the mean TC level was 42 ± 9 mg/dl (min = 29, max = 52; *n* = 5) and increased by at least 95% and at most 299% (163 ± 93%). 7-DHC levels showed a decrease of -28% to -96% (-63 ± 29%). No effect on anthropometric parameters was observed. Overall, the families were satisfied with the care and the effect of the emulsion was predominantly described as successful. The emulsion and its application were well tolerated with few side effects.

**Conclusions:**

Overall, there was an improved effect on TC and 7-DHC levels compared to standard therapy, with high patient satisfaction and low side effects.

## Introduction

Smith-Lemli-Opitz syndrome (SLOS; OMIM: 270400; ICD-11: 5C52.10), also known as 7-dehydrocholesterol reductase deficiency, is a rare autosomal recessive disorder of cholesterol biosynthesis with an incidence of approximately 1:20,000–1:70,000 [1]. The disease was named after the research group of Smith, Lemli and Opitz, who first described the phenotypic characteristics of the disease in 1964 based on three male children. Smith et al. (1964) initially referred to SLOS as RSH syndrome, an acronym derived from the surnames of the first three families identified with the disorder [2]. In 1993, Irons et al. found the first clues to the biochemical basis of the disease in an SLOS patient with unphysiologically low total cholesterol (TC) and elevated 7-dehydrocholesterol (7-DHC) concentrations [3]. Metabolically, SLOS is characterised by reduced activity of the enzyme 7-dehydrocholesterol reductase, which impairs the conversion of 7-DHC to cholesterol [4]. This defect is caused by homozygous or compound heterozygous mutations in the sterol delta 7-reductase gene (DHCR7; 602858; chromosome 11q13) [5]. As 7-DHC serves not only as a precursor for cholesterol biosynthesis but also for vitamin D synthesis, there may be an increase in 25-hydroxycholecalciferol (25-OH vitamin D; 25(OH)D) compared to healthy individuals, with no relevant laboratory or clinical signs of vitamin D toxicity [6].

Pathological levels of TC and 7-DHC in SLO cause characteristic symptoms such as multiple physical malformations and cognitive impairment [7]. This is because cholesterol plays a key role in synthesising steroid hormones, neurosteroids and bile acids, and in formation of cell membranes and supporting embryonic development [1]. Physical features include pre- and postnatal growth restriction, cardiac defects and underdeveloped external genitalia in males. These may be accompanied by postaxial polydactyly, such as the presence of additional fingers/toes on the side of the little finger/toe, and syndactyly of the second and/or third toes. Other symptoms such as constipation, recurrent otitis media, hearing loss, cataracts, strabismus, seizures, limb defects and adrenal insufficiency are common [7]. Those affected often have distinctive facial features: typical features include microcephaly, a flat facial profile, low-set ears, a short nose with an upturned tip, ptosis, epicanthus and retrognathia. These may be accompanied by dental anomalies, wide alveolar ridges and a cleft palate or uvula [8]. Patients with SLOS usually also suffer from intellectual disability due to brain malformations. Most have autistic features, and many are even diagnosed with autism. There may also be hyperactivity, self-injurious behaviour, sleep problems and excessive temperament [1]. Overall, the clinical spectrum of SLO is broad. In addition to severely affected infants whose congenital malformations are so severe that they die in the perinatal period, there are also individuals with minimal physical abnormalities and only mild autistic behaviour [8]. Some malformations occurring in SLOS are associated with a restricted Sonic Hedgehog (Shh) pathway, which plays an important role in developing the central nervous system, facial structures, and limbs [1]. Active Shh proteins are covalently modified with cholesterol, and cholesterol is essential for yielding a protein with a low diffusion rate, the gradients of which are finely regulated during embryogenesis [9]. Therefore, the therapeutic approach to SLOS is limited by the irreversible nature of certain prenatal developmental and structural abnormalities [10].

Dietary treatment of SLOS usually involves cholesterol supplementation to compensate the plasma TC deficiency and to reduce 7-DHC levels via feedback inhibition of 3-hydroxy-3-methylglutaryl coenzyme A reductase (HMG-CoA) [11]. The most common forms of cholesterol supplementation are administration of cholesterol in aqueous or oil-based suspensions or in the form of egg yolk. Typical doses are 30 mg/kg body weight (bw)/day egg yolk and 150 to 300 mg/kg BW/day suspension [11]. Due to its chemical structure, which is both hydrophilic and hydrophobic, cholesterol has low solubility in oil and water [12–14]. Consequently, there is a risk of sediment forming during the preparation of the suspensions, and the cholesterol can also stick together or clump [15]. The administration of cholesterol in the form of egg yolk also has some disadvantages, such as allergies, taste aversions, hygienic problem, and natural variations in the amount of cholesterol contained, which makes a defined dosage difficult [11, 16].

In addition to cholesterol supplementation, some metabolic centres use HMG-CoA reductase inhibitors, such as simvastatin, to decrease 7-DHC levels. It should be noted that simvastatin, as the most lipophilic of the statins, can cross the blood-brain barrier, which could potentially reduce both 7-DHC and cholesterol levels. This could lead to exacerbate cholesterol deficiency, therefore intensive clinical monitoring is required [10]. A recent systematic review concluded that there is currently insufficient evidence to suggest that statins offer any additional benefits to people with SLOS [17].

In this observational study with retrospective data analysis and accompanying structured caregiver interviews, the previous standard therapy was replaced by a cholesterol emulsion in six subjects over an individual period of time with the aim of optimising dietary therapy.

## Materials and methods

### Study population

Patients aged from birth to 18 years with a diagnosis of SLOS, who were regularly treated as outpatients at the University Hospital Münster (UKM) from December 2018, were included. In addition, they had to have previously received standard therapy with non-emulsified cholesterol and have adequate digestive function or no severe intolerance to dietary fats. Inclusion and exclusion criteria are shown in Table 1. Recruitment was based on the medical recommendation of the treating physicians. Caregivers were informed in detail about the background of the study in a personal interview on site and, in case of participation, this was confirmed in writing (written consent). The study was conducted in accordance with the Declaration of Helsinki, and the protocol was approved by the Ethics Committee of the Medical Association of Westphalia-Lippe and the University of Münster (project identification code: 2019-009-f-S). The trial was registered in the German Clinical Trials Register (DRKS; ID: DRKS00015064).

**Table 1:** Inclusion and exclusion criteria for the study population

Inclusion criteria	Exclusion criteria
• Age from birth to 18 years • SLOS (Smith-Lemli-Opitz syndrome) • Previous use of another dietary cholesterol therapy (e.g. in oil, egg yolk, etc.) • Sufficient digestive activity or no severe intolerance to dietary fats	• Other illnesses or metabolic disorders • No previous supplementation of cholesterol • Very critical state of health (e.g. intensive care) • Persistent or repeated vomiting • Serious dietary fat utilisation problems

### Study design and procedure

The study was conducted at the UKM in cooperation with the Center for Nutrition & Therapy (NuT) at the University of Applied Sciences Münster. Based on the dosage of the respective standard cholesterol therapy, an emulsion (see 2.3) was prescribed in a dose-equivalent manner and the dosage was adjusted to the needs of the respective patient during the course of therapy. In order to improve tolerability and avoid side effects, the amount of emulsion was slowly increased individually. Patients received either a tube-fed or oral formulation of the developed emulsion, depending on their individual needs and current diet. On prescription, the emulsion was prepared by a pharmacy chosen by the patient using the standard formulation (see Table 2). The total amount of emulsion was administered individually throughout the day. To achieve a suitable consistency for administration, the emulsion should be removed from the refrigerator at least 30 min before use. The emulsion developed for oral administration could be consumed directly or mixed with other foods such as yoghurt, pudding or fruit puree. Apart from possible adjustments to existing dietary plans, such as reducing fat intake from accompanying foods, no other changes were required for patients. Blood samples for triglycerides (TG), cholesterol (TC, low-density lipoprotein [LDL], high-density lipoprotein [HDL], 7-DHC) and vitamin D (1,25(OH)_2_D, 25(OH)D) were taken at regular follow-up visits to minimise patient burden. The outpatient appointments varied according to the patient’s health and family situation. The patients’ height and weight were recorded during the follow-up visits.

### Preparing of the cholesterol emulsion

The components of the developed emulsion are cholesterol, vegetable oil, solid edible fat, water and optionally medium-chain triglyceride (MCT) oil. The emulsion does not contain any other additives, but can be variably adapted by adding, for example, minerals, vitamins, sweeteners, flavourings, colourings, etc. The gastric and jejunal tube compatible variants have a lower viscosity, lower cholesterol content (2–5% w/w) and additional MCT compared to the oral variants. The oral variants do not contain MCT oil and the cholesterol content is higher (5–25% w/w) due to the significant influence of cholesterol on the viscosity of the final product. The standard formulation for the oral emulsion contains 2 g of cholesterol per approximately 20 ml of emulsion (8–9%; w/w; ~100 mg/mL) and the version for administration by tube contains 0.5 g of cholesterol per approximately 20 ml of emulsion (2–3%; w/w; ~25 mg/mL) with the same weight ratio of water to total fat (see Table 1). Finished standard emulsions can be stored in the refrigerator at about + 4 °C for at least four weeks. Outside the refrigerator, at room temperature, they are stable for a maximum of 24 h. Further information on the development of the emulsions can be found in the patent application DE102018112898A1 [15].

**Table 2:** Standard formulation of tube-compatible (0.5 g) and orally ingestible (2.0 g) cholesterol emulsions for use in patients with SLOS

	tube-compatible	oral
Cholesterol	0.5 g (~ 25 mg/mL)	2.0 g (~ 100 mg/mL)
Vegetable Oil (e.g. rapeseed oil)	5.0 mL	7.5 mL
Solid fat (e.g. coconut fat)	2.5 mL	2.5 mL
MCT oil	2.5 mL	0 mL
Water	10.0 mL	10.0 mL

### Structured caregiver interview

Caregivers’ satisfaction with care, diet, use of emulsions, and subjective perceptions of their behaviour and health were assessed using structured telephone interviews. The questionnaire was developed and modified based on the questionnaire ‘How are you coping with your PKU at the moment’, developed by Prof. Karin Lange and PD Dr. Gundula Ernst, Medical Psychology, Hannover Medical University [18]. The 30-minute interviews were conducted in April 2024 by a trained nutrition scientist. A total of 46 questions were asked, consisting of closed, open and combined questions. In addition to dichotomous, linked and open-ended response formats, the interview guide included response options with a five-point unipolar rating scale with verbal scale labelling (see Appendix 1). The different question formats were designed to elicit responses that were as comprehensive as possible but still comparable.

### Sampling and analysis

To determine the difference between the previous standard therapy and the emulsion, a retrospective evaluation of existing clinical patient data from the UKM from inpatient and outpatient stays was carried out. In addition, blood values documented in the medical records by external laboratories or requested from the families were used. Data were extracted from the ORBIS clinical software (Dedalus Healthcare; Bonn, Germany) or from paper-based clinical reports (see 2.6).

TC, HDL, and LDL in human plasma were measured by using enzymatic assays with the automated clinical chemistry analyzer Cobas c502 (Roche Diagnostics GmbH, Mannheim, Germany) according to the standard procedure recommended by the manufacturer. 7-DHC was measured with gas chromatography according to Seedorf et al., 1995 [19]. The levels of 25(OH)D and 1,25(OH)_2_D were assessed by chemiluminescence immunoassay kit LIAISON^®^ Vitamin D assay on a LIAISON^®^ XL (Diasorin Deutschland GmbH, Dietzenbach, Germany). The methods were validated by regular analyses of reference sera supplied by the national German INSTAND proficiency testing program and the international quality assurance program of the US Centers for Disease Control and Prevention.

### Sample size and statistical analysis

All available data were transferred to Microsoft Excel 365 and categorised into annual intervals (t_0_-t_7_). The mean values of the last two years before the change in therapy were used as baseline values (t_0_). Analysis of the structured interview included frequency analysis and summary of responses. Body mass index (BMI; kg/m²) was calculated from the anthropometric data of height (m) and weight (kg). The anthropometric data were compared with the national reference percentiles according to Kromeyer-Hauschild et al. [20, 21] and the SLOS growth charts from Lee et al. [22]. All quantitative data were first analysed using descriptive statistics such as minimum, maximum, mean, median and standard deviation (SD). IBM SPSS Statistics 29 was used for advanced data processing.

Kolmogorov-Smirnov test, Shapiro-Wilk test and graphical analysis were used to assess normal distribution. For normally distributed parameters (TC, 7-DHC, HDL, LDL), one-way analysis of variance (ANOVA) with repeated measures was used. In the case of non-normal distribution (25(OH)D), the non-parametric Friedman test was used. Due to the limited amount of data, statistical testing was not possible for the parameter 1,25(OH)_2_D. In repeated measures ANOVA, a Greenhouse-Geisser correction for degrees of freedom was applied to all tests where the Mauchly sphericity test failed. In addition, a Bonferroni-corrected post-hoc test was performed to identify differences between measurement times. In the ANOVA, the effect size was determined for all results using a paired t-test and for the Friedman test using Kendall’s W-test. The significance level was *p* < 0.05. Due to the small sample size, gender was not considered in the analysis.

To detect a clinically relevant change in the main outcome variable TC (mg/dL; t_0_ = 40, t_max_ = 100, SD = 30; correlation between t_0_ and t_max_ = 0; effect size dz = 1.644) with 90% power and a two-sided alpha of 0.05, 5 participants were required. In case of dropouts, two additional participants (30%; total = 7) were recruited. Sample size was calculated using the scientific freeware G∗ Power, version 3.1.9.2 (critical t = 2.131; calculated power = 0.916).

## Results

A total of seven patients (m = 6, f = 1) were recruited for the study and received the cholesterol emulsion. One subject was excluded from the data analysis because of poor compliance during the study, which meant that it could not be assumed that the emulsion would be administered regularly. The remaining six subjects (m = 5, f = 1; 4.8 ± 4.4 years; Md = 2 years; min = 1 year, max = 12 years) received an individualised dose of cholesterol in oil as standard therapy at 149–205 mg/kg bw/day for at least one year (max = 12 years) before the cholesterol emulsion was administered. The first participant was switched to the emulsion in June 2017 and the last participant in July 2019. The total duration of use of the cholesterol emulsion was 4.2 ± 2.5 years (Md = 3.5; min = 2, max = 7). Patients received 16–200 mL of the emulsion daily, corresponding to 1.6–5 g cholesterol/d and an average of 160 ± 55 mg/kg bw/day (min = 80, max = 282 mg/kg bw/day), which varied intra- and interindividually during the intervention period.

### TC in plasma

**Fig. 1:**
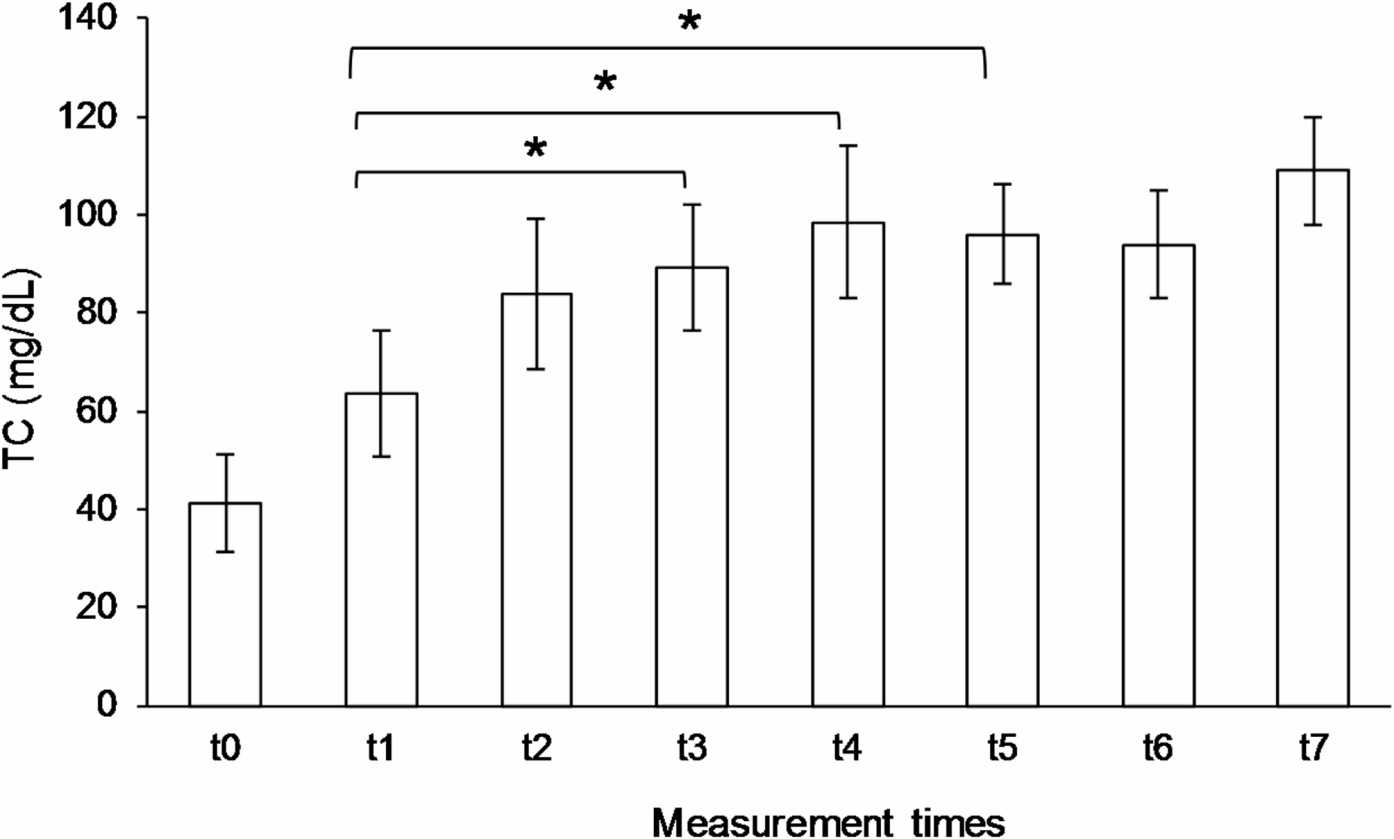
Means and standard deviations of TC plasma levels at time points t_0_-t_5_ (*n* = 4), t_6_ (*n* = 3) and t_7_ (*n* = 2). Comparison of t_1_ vs. t_3_ (*p* = 0.030), t_1_ vs. t_4_ (*p* = 0.033) and t_1_ vs. t_5_ (*p* = 0.031) showed significant differences (*)

The average plasma TC level (t_0_ = 42 ± 9 mg/dL; Md = 45; min = 29, max = 52; *n* = 5) of the patients increased continuously while taking the emulsion, with strong individual differences. After one year (t_1_) of therapy with the emulsion, the cholesterol level increased to a mean of 63 ± 11 mg/dL (min = 50, max = 75, *n* = 5, *p* = 0.197), after two years (t_2_) to 76 ± 22 mg/dL (min = 44, max = 104, *n* = 5, *p* = 0.173), after three years (t_3_) to 89 ± 13 mg/dL (min = 75, max = 102, *n* = 4, *p* = 0. 198), after four years (t_4_) to 99 ± 16 mg/dL (min = 86, max = 119, *n* = 4, *p* = 0.146), after five years (t_5_) to 96 ± 10 mg/dL (min = 85, max = 109, *n* = 4, *p* = 0.1 63), after six years (t_6_) to 94 ± 11 mg/dL (min = 83, max = 105, *n* = 3) and after seven years (t_7_) to 109 ± 1 mg/dL (min = 101, max = 117, *n* = 2). The Bonferroni-corrected post-hoc test (*n* = 4) showed a significant increase in TC levels from t_1_ to t_3_ (*p* = 0.030), to t_4_ (*p* = 0.033) and to t_5_ (*p* = 0.031) (see Fig. 1). In 80% (*n* = 4) of the subjects evaluated, TC levels increased by at least 95% and a maximum of 299% (163 ± 93%) from t_0_ to the last year of intervention.

### 7-DHC in plasma

**Fig. 2:**
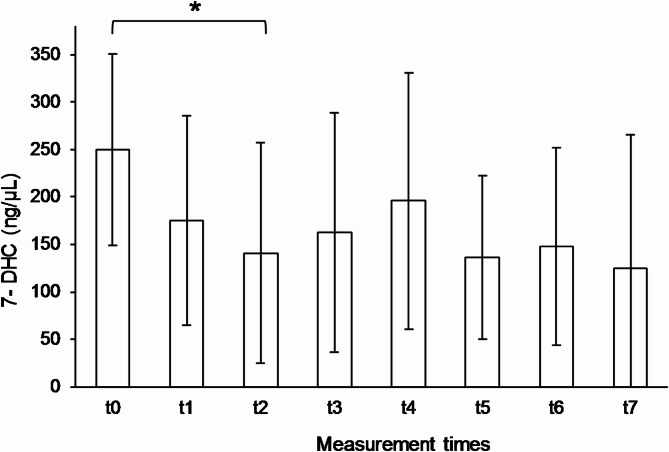
Means and standard deviations of 7-DHC plasma levels at time points t_0_-t_2_ (*n* = 6), t_3_-t_5_ (*n* = 4), t_6_ (*n* = 3) and t_7_ (*n* = 2). The comparison of t_0_ vs. t_2_ (*p* = 0.011) showed a significant difference (*)

At baseline (t_0_), the average 7-DHC level was 250 ± 101 ng/µL (min = 149, max = 408, *n* = 6). At time point t_1_, 7-DHC levels decreased to 175 ± 110 ng/µL (min = 19, max = 319, *n* = 6, *p* = 0.291). In the further course a value of 141 ± 116 ng/µL (min = 9, max = 294, *n* = 6, *p* = 0.011) was observed at t_2_, t_3_ of 163 ± 126 ng/µL (min = 49, max = 274, *n* = 4, *p* = 0.100), t_4_ of 196 ± 135 ng/µL (min = 62, max = 367, *n* = 4, *p* = 0. 234), t_5_ of 136 ± 86 ng/µL (min = 33, max = 239, *n* = 4, *p* = 0.060), t_6_ of 148 ± 104 ng/µL (min = 28, max = 209, *n* = 3) and t_7_ of 125 ± 140 ng/µL (min = 26, max = 224, *n* = 2). A statistically significant difference was observed between t_0_ and t_2_ (*p* = 0.011; see Fig. 2). In all subjects, the 7-DHC level decreased by at least 28%, with a maximum reduction of 96% (M = −63 ± 29%) from t_0_ to the last year of the intervention.

### HDL and LDL cholesterol in plasma

Plasma HDL levels showed strong inter-individual variability. From t_0_, there was an increase of at least 30% and a maximum increase of 106% by the end of the observation period (M = 77 ± 36%). At t_0_, HDL during the previous standard therapy was 26 ± 7 mg/dL (SD = 7, min = 18, max = 37, *n* = 5) and subsequently increased to 44 ± 12 mg/dL (t_5_; min = 33, max = 57, *n* = 3) and 46 ± 14 mg/dL (t_6_; min = 37, max = 62, *n* = 3). LDL increased by a minimum of 20% and a maximum of 159% (M = 67 ± 65%). The initial value at t_0_ was 25 ± 8 mg/dL (min = 16, max = 34, *n* = 4) and reached mean values of 39 ± 4 mg/dL (t_6_; min = 34, max = 42, *n* = 3) and 43 ± 3 mg/dL (t_5_; min = 41, max = 46, *n* = 3). In individual cases, LDL plasma values of 49 mg/dL (t_7_) and 50 mg/dL (t_4_) were determined.

### 25-(OH)- and 1,25(OH)2-Cholecalciferol in plasma

25(OH)D was 62 ± 39 ng/mL (min = 34, max = 90, *n* = 2) during the previous standard therapy (t_0_) and 41 ± 9 ng/mL (min = 36, max = 51, *n* = 5) at t_1_. Further, there was a slight increase up to 69.8 ng/mL (t_3_) with high individual variability. The mean 1,25(OH)_2_D was 58 ± 6 pg/mL at t_0_ (min = 54, max = 62, *n* = 2). At t_1_ the 1,25(OH)_2_D level was 70 ± 23 pg/mL (min = 48, max = 62, *n* = 5), t_2_ 55 ± 19 pg/mL (min = 37, max = 82, *n* = 4), t_3_ 64 ± 1 8 pg/mL (min = 40, max = 78, *n* = 4), t_4_ at 58 ± 24 pg/mL (min = 41, max = 75, *n* = 2) and t_5_ at 62 ± 25 pg/mL (min = 44, max = 80, *n* = 2). For t_6_ and t_7_, only values from one subject could be determined.

### Anthropometric measurements

According to the national reference percentiles 67% (*n* = 4) of patients were below the 50th percentile for both weight and height, before and after the change in therapy. Individually, there was a tendency to fall below or remain stable at the 3rd percentile for weight (67%), height (67%) and BMI (50%). Thus, in the majority of cases, there is a reduction in growth with a corresponding reduction in weight. In one case, a BMI above the 99.5th percentile was due to a combination of low height growth (< 3rd percentile) with a concomitant weight around the 50th percentile. In another case, there was a large fluctuation in BMI from P50 to P90 during the observation period. Using the SLOS growth charts, there was a tendency to fall below or remain within the 5th percentile for weight in 33% of cases (*n* = 2), for height in 16.6% of cases (*n* = 1), and for BMI in 50% of cases (*n* = 3). Two cases (33.3%) exceeded the 95th percentile for BMI. Overall, no clear anthropometric changes were observed after the change in therapy.

### Structured caregiver interview on therapy satisfaction

In total, the carers of five patients (m = 4, f = 1) participated in the additional structured interview. The telephone interview lasted 21–30 min (Ø 27 min). Overall, the therapeutic approach with the cholesterol emulsion was well understood (80%, *n* = 4) and the medical care was largely perceived as positive. At the time of the interview, all patients were taking the cholesterol emulsion regularly (3.8–5.5 g cholesterol/d; 4.5 ± 0.6 g/d). The daily dose was divided into two portions in one patient, four portions in three patients and six portions in one patient. Three patients took the enterally administered emulsion, one patient took the orally administered emulsion and one patient took an individually formulated emulsion. Of the two subjects taking the oral formulation, one received the emulsion mixed with milk as a bottle feed and the other received the emulsion mixed with his regular pureed food. The majority of patients (80%, *n* = 4) reported good daily use. Only in two cases were occasional uneven mixing of the emulsion and deposits on the bottle wall reported. The tolerability of the emulsion was described as good by all respondents. Three persons described side effects, one of whom reported positive side effects such as better and softer bowel movements and less constipation. At the beginning of the therapy, one subject reacted with initial vomiting and another with diarrhoea. None of the subjects reported persistent vomiting, constipation or bloating. The success of the cholesterol emulsion was rated as ‘no success’ by one respondent (20%), ‘moderate success’ by another (20%) and ‘great success’ or ‘very great success’ by three respondents (60%). The child’s current TC levels were rated as satisfactory in the majority of cases (60%). In the other two cases, the levels were either unknown or not considered important. Three of the respondents (60%) noticed a change in their child’s behaviour while the emulsion was being administered. While one reported that the child seemed more attentive and focused, participated more actively, and had better sleep behaviour, another reported a happier and more positive child who had shown developmental progress since starting the emulsion. In no case was a negative change in behaviour directly attributed to the cholesterol emulsion. Table 3 summarises the main findings of the structured interview.

**Table 3:** Percentage results with information on the frequencies (n) of the structured caregiver interview on therapy satisfaction, presenting the main aspects of the subtopics of care satisfaction, nutrition, emulsion use and subjective behaviour change

**Care satisfaction**	
Good knowledge of the disease	100% (*n* = 5)
Good medical care	80% (*n* = 4)
Easy to understand explanation of the disease	80% (*n* = 4)
**Nutrition**	
Enteral nutrition	60% (*n* = 3)
No change in eating behaviour due to emulsion	100% (*n* = 5)
Dietary adjustment after change to emulsion (lower fat)	40% (*n* = 2)
**Emulsion use**	
Understandable therapeutic approach	80% (*n* = 4)
Easy to use	80% (*n* = 4)
Well tolerated	100% (*n* = 5)
Side effects	60% (*n* = 3)
No weight change	80% (*n* = 4)
***Satisfaction with cholesterol levels***	
Not relevant	20% (*n* = 1)
Levels unknown	20% (*n* = 1)
Satisfied	60% (*n* = 3)
***Evaluation of success***	
No success	20% (*n* = 1)
Moderate success	20% (*n* = 1)
Great to very great success	60% (*n* = 3)
**Subjective behaviour change**	
Behavioural changes after emulsion administration	60% (*n* = 3)
Of the reported changes: neutral or beneficial changes	100% (*n* = 3)

## Discussion

The present observational study is the first to investigate the use of a cholesterol emulsion in SLOS. In all cases, an increase in plasma TC levels of at least 95% and at most 299% (163 ± 93%) was observed, with a concomitant decrease in 7-DHC levels of −28% to −96% (M = −63 ± 29%). This is consistent with the results of other trials using cholesterol supplementation [23, 24]. In this context, it is suggested that higher cholesterol levels increase feedback inhibition, thereby reducing the toxic metabolites 7-DHC and 8-DHC [11]. Compared with the previous cholesterol supplementation or the results of other studies in SLOS, the emulsion showed a better or equivalent effect on TC and 7-DHC than with the administration of egg yolk or crystalline cholesterol in oil. Linck et al. (2000) observed an increase in mean plasma cholesterol from 53 to 82 mg/dL (+ 56%) after 4 to 8 weeks and a further increase to an average of 115 mg/dL (+ 116%) after 35 weeks in four patients with SLOS who were given egg yolk (18–60 mg cholesterol/kg/day). At the same time, a 67% reduction in 7-DHC from 10.7 to 3.5 mg/dL was observed after 35 weeks [23]. Nwokoro and Mulvihill (1997) also found an increase in plasma cholesterol with cholesterol replacement therapy in a case series of six patients. However, 7-DHC did not change significantly during the observation period [25]. In a multicentre study by Irons et al. (1997) with a total of 14 patients and a treatment duration of 6–15 months, 3 patients received cholesterol supplementation (40–120 mg cholesterol/kg/day) with and without the addition of one or more bile acids, and 9 patients received no supplementation. In two cases, the cholesterol was given in the form of egg yolk, and in the remaining patients, the cholesterol was administered as a suspension. On average, TC increased by 131% (max. 235%) with bile acid supplementation and by 163.5% (max. 238.7) without bile acid supplementation. The comparatively high increase in the group (*n* = 3) without bile acid supplementation is mainly due to one patient who showed a change of 416.7%. The other two patients in this group only showed an increase in TC of 13.2 and 60.6%. Overall, the addition of bile acid did not appear to have any additional beneficial effect on TC levels. The results were not separated according to the different forms of administration, i.e. egg yolk or suspension. In addition, most patients who started with low TC levels remained below 100 mg/dL [26]. With regard to the administration of egg yolk, it should be noted that the studies used an average cholesterol content of 210 mg per egg yolk, which cannot be standardised [23, 26]. This is because the cholesterol content of eggs depends on many factors, such as the size of the egg, the breed, the age of the hen or the diet of the hens, which means that natural variations are unavoidable [27–29]. Consequently, the exact amount of cholesterol ingested when egg yolk is administered cannot be accurately determined. It also requires a lot of preparation, as the egg white must be removed in most cases to avoid allergies [23, 26]. Linck et al. 2000 justify the use of egg yolk on the basis that cholesterol is not sufficiently soluble in oil and therefore absorption is expected to be low [23]. In this context, it was found in 11 SLOS patients that, based on test meals supplemented with radiolabelled cholesterol-4-C^14^, cholesterol absorption was 27.3 ± 6.7% for egg yolk and 20.5 ± 10.3% for crystalline cholesterol. Irrespective of the source of cholesterol, cholesterol absorption appears to be impaired in SLOS, to a lesser extent than in healthy adults or hypercholesterolaemic children. Reduced bile acid synthesis or competing effects of 7-DHC are thought to be the cause of impaired cholesterol absorption [30]. The steady increase in TC observed over the years is probably due to adjustments in the dosage of the cholesterol emulsion relating to the patients’ physiological development.

In terms of clinical effects, improved growth, better development and fewer behavioural problems have been described as a result of cholesterol supplementation in SLOS [25, 26, 31]. Positive effects on puberty, reduced susceptibility to infection, reduced photosensitivity and skin rashes have also been reported [25, 31]. Subjective observations by relatives and others in the patients’ environment, such as teachers and therapists, showed that the children were more active and happier, and that there was a general feeling of improvement compared with the time before supplementation started [26, 31]. This is consistent with statements from the structured interview, in which the majority (60%) of carers interviewed associated an improvement in behaviour with the emulsion used. This is contradicted by a study by Sikora et al. (2004), who critically reviewed anecdotal reports of developmental improvement in SLOS patients in relation to cholesterol supplementation, and found no measurable change at any developmental level in a 6-year longitudinal study [24]. In particular, perceived behavioural changes contradict the knowledge that cholesterol cannot cross the blood-brain barrier. In this context, Elias et al. (1997) suggest that severe cholesterol deficiency increases the permeability of the blood-brain barrier to cholesterol [26]. In contrast, there are results showing no increase in cerebrospinal fluid (CSF) cholesterol following supplementation [32, 33]. Whether or not dietary cholesterol crosses the blood-brain barrier, some patients with SLOS have developmental brain abnormalities that are irreversible and therefore cannot be treated by diet [11]. The rapid behavioural changes described are explained in part by a ‘sterol effect’, whereby cholesterol becomes available again as a starting point for steroid production [26]. A positive influence on puberty and the development of the phallus can thus be justified by an increased synthesis of sex hormones [31, 34]. There is also evidence that cholesterol supplementation may improve photosensitivity in patients [35]. However, although it has not been scientifically proven that cholesterol supplementation leads to a clear improvement in the development of patients with SLOS, other positive effects have been observed that contribute to an improved quality of life and support the use of cholesterol supplementation.

There was no improvement in growth after the patients were adjusted to the cholesterol emulsion. However, it should be noted that all the patients were already receiving a cholesterol supplement, so no direct before-and-after effect would be expected. In all cases, there were no serious negative or positive effects on height or weight development. Reduced growth is well known in patients with SLOS and has been described in longitudinal data from 78 patients, with a growth delay of two standard deviations compared with age-matched children. The same has been found for BMI, which does not apply to the patient population described here due to the presence of overweight and obesity. The deviations between the results of the national reference percentiles and the SLOS growth charts are in line with expectations, given the growth retardation reported by Lee et al. [22].

Levels of 25(OH)D, HDL and LDL were not significantly increased. No statistical test could be performed for 1,25(OH)2D due to missing data. Patients’ vitamin D levels were consistently in the sufficient, non-toxic range [36]. There was no trend towards an increase or decrease as a result of the intervention. In a retrospective analysis of medical records, Movassaghi et al. 2017 reported significantly higher 25(OH)D levels in patients with SLOS (48.06 ± 19.53 ng/mL) in all seasons compared to patients without SLOS (30.51 ± 16.14 ng/mL), but the levels in both groups were in the middle of the reference ranges considered sufficient by the Endocrine Society and not in the toxic range [6, 36]. As 7-DHC is the precursor of 25(OH)D, elevated levels are comprehensible. However, it should be noted that the ability to synthesise depends on UV exposure, which may be low due to limited sunlight exposure, depending on the severity of the disease [37, 38].

In standard dietary therapy for SLOS, cholesterol is considered safe, with few side effects and is well tolerated [7]. None of the available trials of cholesterol supplementation in SLOS reported side effects [23, 26, 31]. This was confirmed when cholesterol was administered in the form of an emulsion. All carers described the emulsion as well tolerated and the administration of the emulsion was associated with a few mild side effects. The side effects experienced by two patients (initial vomiting, diarrhoea) were in line with the usual problems associated with a change of diet and can be explained by the simultaneous high intake of dietary fats. In addition, diarrhoea only occurred in one patient with a pre-existing Clostridioides difficile infection, so no direct conclusion can be drawn about the emulsion. A positive side effect of the cholesterol emulsion was a softening of the stool, which counteracted the typical SLOS symptom of constipation.

According to the data presented, the cholesterol emulsion has a good enteral absorption capacity due to its fine distribution, which increases the resorptive surface. In addition, the emulsion allows for optimal dosage adjustment, which is not the case with egg yolk, for example. Furthermore, the hydrophilic and hydrophobic components of the emulsion provide a patient-focused cholesterol emulsion through the addition of vitamins, minerals, flavours, sweeteners or a targeted selection of fats, e.g. omega-3 fatty acids [15]. Unlike commercially available products, the emulsion does not contain added sugars such as maltodextrin, glucose, etc., which are unnecessary for use in SLOS and do not contribute to improved absorption.

The administration of the cholesterol emulsion is associated with an additional intake of fat, which may lead to an increased energy intake. In the present study, one patient’s diet had to be adjusted because of pre-existing overweight. This should be done by a dietician who closely monitors the patient’s weight and adjusts the diet.

The present study has some limitations, such as the lack of a control group and the overall small sample size, which does not allow for subgroup analyses, such as gender differences. In addition, there was considerable individual variation in prescribed cholesterol doses and, because the data were collected during routine outpatient appointments, there was a high degree of variation in the time of measurement. There were also occasional gaps in the data, mainly due to staff turnover and documentation problems. Due to the long duration of the study (more than 4 years), the varying severity of the disease, the high heterogeneity of the disease pattern, and the small number of patients, which is severely limited by the low incidence of the disease, these problems in the clinical setting can only be avoided to a limited extent. The study design was chosen to minimise the burden on patients while providing a realistic picture of usual therapy.

## Conclusion

The present long-term observational study is the first to investigate cholesterol supplementation as an emulsion for SLOS and provides important insights into its practical application and influence on metabolic parameters. Overall, an improved effect on TC and 7-DHC levels was observed compared with standard therapy. The results of the structured interview showed a high level of user satisfaction and highlighted the potential of the new dosage form. Overall, the cholesterol emulsion can be described as well tolerated and with few side effects, while at the same time offering the possibility of individual patient adjustment. The optimised cholesterol dosage form thus represents a potential alternative to the current standard therapy.

## Data Availability

The datasets used and/or analysed during the current study available from the corresponding author on reasonable request.
